# Mid-Frontal Theta Modulates Response Inhibition and Decision Making Processes in Emotional Contexts

**DOI:** 10.3390/brainsci9100271

**Published:** 2019-10-11

**Authors:** Siddharth Nayak, ChiiShyang Kuo, Arthur Chih-Hsin Tsai

**Affiliations:** 1 Taiwan International Graduate Program in Interdisciplinary Neuroscience, National Cheng Kung University and Academia Sinica, Taipei 11529, Taiwan; 2 Department of Electrical Engineering, National Central University, Chungli, Zhongli District, Taoyuan City 32001, Taiwan; 3 Institute of Statistical Science, Academia Sinica, Taipei 11529, Taiwan

**Keywords:** event-related spectral perturbation (ERSP), theta, beta, response inhibition, stop signal, hierarchical drift diffusion modeling (HDDM)

## Abstract

Inhibitory control is an integral part of executive functions. In this study, we report event-related spectral perturbation (ERSP) results from 15 healthy adults performing an emotional stop-signal task with the use of happy, disgusted, and neutral emotional faces. Our ERSP results at the group level suggest that changes in low frequency oscillatory power for emotional and neutral conditions start at as early as 200 ms after stimulus onset and 300 ms before button press for successful go trials. To quantify the dynamics of trial-by-trial theta power, we applied the hierarchical drift diffusion model to single-trial ERSP at the mid-frontal electrode site for the go condition. Hierarchical drift diffusion modeling (HDDM) assigned higher frontal low-frequency oscillatory power for evidence accumulation in emotional contexts as compared to a neutral setting. Our results provide new evidence for dynamic modulation of sensory processing of go stimuli in inhibition and extend our knowledge for processing of response inhibition in emotional contexts.

## 1. Introduction

Inhibitory control is a critical component of executive function measured in tasks where participants typically have to inhibit a prepotent response [1]. It is best measured in the laboratory in the form of a go/no-go task or stop-signal paradigm. In the stop-signal paradigm, participants make simple choice decisions about a visual stimulus, but in some random trials, a signal is present after the stimulus, indicating that the response is withheld. Behavioral measures of inhibitory control are calculated using the stop-signal reaction time (SSRT), an index to measure how long it takes an individual to inhibit a response. 

A lot of studies have focused on mechanisms activated by the stop-stimulus, popularly referred to as reactive inhibition [2]. Reactive inhibition deals with stopping of motor response which is already in progress. A network of brain regions for this reactive inhibition (also commonly referred to as motor inhibition) process recruits the right inferior frontal cortex (rIFC), the pre-supplementary motor area (pre-SMA), and the subthalamic nucleus (STN) [3,4,5]. These brain regions are deemed to be activated by the stop stimulus. A slow reaction time for the go stimulus enhances the likelihood of successful inhibition for a particular trial, and sensory processes involved with the stop stimulus are mostly assumed to influence reactive inhibition [6,7]. 

Previous studies have shown that reaction times can be adjusted at a very short interval [8] and could be probably related to the calculation of the trial-wise probability of coming across a stop signal in a future trial [9]. Besides, experimental studies converge on the evidence that proactive and reactive inhibition might recruit similar brain networks for inhibitory processing [5]. However, proactive inhibition imparts a partial selective inhibition instead of a complete stopping [10,11]. A typical assumption is that proactive and reactive inhibitory behavior is contingent on the accomplishment of a vital response inhibition component [12]. However, formal modeling suggests that the enormous proportion of time required to process response inhibition is engaged by sensory and motor processes relating to the treatment of the stop stimulus [13,14,15] and recently published work has focused on modifying inhibition burdens by adjusting for such kind of methods [16,17]. The speed-accuracy tradeoff associated with fast decision making can be mathematically realized by an increasing or decreasing decision threshold, that is, a measure which defines when the uninterrupted accumulation of data concludes, and the option with the robust evidence is chosen [18,19]. The basal ganglia (BG) regions are associated with neurobiological models of decision making underlying speed-accuracy tradeoffs, due to their wide range of connections to cortical and subcortical areas engaged in movement- and decision-related processes [20]. The BG exert tonic inhibition over cortical regions, which can be diminished through stimulation of a direct pathway between the striatum with BG output areas or amplified by triggering of two inhibitory circuits passing through the subthalamic nucleus [3], which integrate to form the indirect and hyper-direct pathways [21].

While the involvement of sensory processes in response inhibition has received some attention [22], there is a lot of support with respect to the role of early attentional processes in emotional perceptual tasks [23] as well as their interaction [24]. Affective stimuli show prioritized perceptual processing [3,25,26,27]. Previous reports on emotional content on response inhibition have mixed results, with emotional stimuli having an improvement, hindrance, or no significant effect on the behavioral performance of response inhibition. These variable results can be explained in part by the dual competition framework [25]. This framework posits that emotion affects perceptual and executive control. When an emotional stimulus is low in threat, then it improves task performance and promotes goal-directed behavior. The perceptual process of the emotional stimuli attracts attention, thereby improving executive control as referenced in response inhibition in terms of higher accuracy for no-go trials [28] and shorter stop-signal reaction times [29,30]. On the other hand, when an emotional stimulus is high in threat, it then impairs task performance and effects goal-directed behavior by competing with executive control for attentional resources, as is evident from reduced accuracy for no-go trials [31] and longer stop-signal reaction times [30,32].

Time-frequency analyses in the human scalp electroencephalogram (EEG) display a marker of successful inhibition at frontocentral scalp locations, explicitly in the delta- (1–4 Hz) and theta- (4–8 Hz) frequency bands [10,33,34,35]. A recent review on the topic of response inhibition found that the current role of oscillatory processes involved in human EEGs is still ambiguous and requires further understanding [36]. A common finding from no-go or stop trials is amplified theta and delta oscillatory power during 200–600 ms post stop stimulus onset [34,37,38,39]. Intracranial EEG recordings in the primary motor cortex (M1) and the right inferior frontal gyrus (IFG) have established inhibition-related enhancement in beta oscillatory power [40], indicating another source of discrepancy in the time-frequency domain, possibly causative of inhibitory processes. Finally, deep brain stimulation (DBS) of the STN improves stopping behavior marked by the beta-band activity over the right frontal cortex [41], providing support to the role of the fronto-basal-ganglia circuit in preventing an initiated response.

Electrophysiological recordings of STN local field potential (LFP) and EEGs have revealed that low frequency oscillatory (LFO) power activations during cognitive–motor tasks are primarily related to prefrontal cortex (PFC) activity and PFC–STN connectivity [42,43,44,45]. Changes in cortical LFO power were found to be localized mainly to medial PFC measured by scalp EEG in non-clinical subjects performing a speed-accuracy tradeoff task [46]. A recent study revealed that trial-by -trial differences of Fz electrodes in LFO power measured using EEG were associated with the activity of medial PFC measured using functional magnetic resonance imaging (fMRI) and that both signals were related to an escalation in decision thresholds. These findings add to the growing literature of the role of fronto-subthalamic circuits in decision making and modulations of the speed–accuracy trade-off [21,47]. The medial prefrontal cortex has structural connections to the STN via the indirect pathway and the direct pathway [20,48,49]. The PFC heightens its influence over STN when task complexity increases [50] leading to amplified decision thresholds to postpone the response [44,46]. Previous studies accessing conflict using the Flanker task reported amplified STN LFO power in trials with task-irrelevant stimuli compared to trials having task-relevant stimulus [51,52,53]. Response conflict is associated with increased decision thresholds [54]; the reported decision threshold changes are correlated to LFO modifications. However, it would be premature to conclude that LFO activity recorded in exploratory EEG studies over the PFC would simply map to one precise mechanism, as it has been shown previously to be not only modified by conflict but also several other processes including emotional reactivity, punishment, novelty, error, memory, and learning [42,55]. 

We can define proactive inhibition by delayed response times in situations where outright stopping (or reactive inhibitory control) might be required. A late response to go stimuli increases the probability of successful stopping for any given trial, and these preparatory processes help in reactive inhibition. Recent work in this domain has suggested the role of attentional processes influencing go-stimulus processing [56,57,58]. Electroencephalographic (EEG) studies have provided evidence for the inferoposterior N1 component (an index of selective attentional processing) being down-regulated as response times were slower, but only when outright stopping was contextually relevant [57,58], suggesting that proactive inhibition reflects attentional processes which are selective rather than being related to global stopping, as measured by reactive inhibition which focusses on stop trials. Another reason to focus on the go stimulus rather than on the stop stimulus is that performance in stop-signal tasks is determined by other aspects of the decision process as well, including the level of caution in responding and the speed of processing the go stimulus. These aspects cannot be measured by fixating on stop trials only and measuring SSRTs from stop trials. To throw more light on these aspects of decision making during response inhibition, we focused on the drift-diffusion model to tease apart stop-signal task data into psychologically essential components.

The current study used EEG analyses to elucidate the role of frontal theta power in emotional response inhibition. We concentrated on go trials and their association with the sensory processes in the early time window and reaction time in emotional-stop (ES) and neutral-stop (NS) conditions since medial frontal LFO power has been shown to contribute to elevated decision thresholds during different decision-making processes. The swift sequential progression of go and stop stimuli is particularly relevant here since this would prevent an exact emotional effect on stop stimuli without concurrently also improving the visual processes involved with go stimuli presented at the identical spatial position. In this study, we present a go stimulus and stop stimulus at the same spatial location on the computer screen; it would impact the processing of affective stop stimuli when go stimuli are also loaded with affect since the processing of the go stimulus is not complete when the stop stimulus appears on the screen. The association between reaction time (RT) and inhibitory performance in a drift-diffusion model has been demonstrated previously in a few studies [58,59,60]. Specifically, we were intent to understand whether (1) evidence buildup would progress sooner in an emotional context, and (2) medial frontal LFO/beta power modulated the trial-by-trial decision-making process for ES in comparison with NS condition, which would be in line with a sensory process increase in response to go stimuli. For the ES condition, we hypothesized two different sample of results: (1) The association between trial-by-trial mid-frontal LFO/beta power and decision-making processes across ES conditions would be more pronounced than across NS conditions; (2) The overall attentional escalations driven by an emotional context [61,62,63] might engage medial frontal LFO/beta power to disrupt the association between visual processes and the decision threshold. To test our specific hypotheses, we modeled emotional stop-signal data as an evidence accumulation process to pick apart the perceptual decision making dimensions that are relevant for differentiating between the ES and NS conditions. In addition to this, we modeled medial frontal theta and beta power variation in a trial-by-trial fashion in a drift-diffusion model to tease apart emotional context influences on medial frontal theta or beta activity on decision threshold.

## 2. Materials and Methods

### 2.1. Participants 

Seventeen volunteers participated in this study. All participants were right-handed and had normal vision with no history of psychiatric illness or mental disorder. All methods were carried out following relevant guidelines and regulations with the Human Research Ethics Committee IRB on Biomedical Science Research/IRB-BM Academia Sinica, Taiwan with the approval number AS-IRB-BM-13058. None of the subjects claimed to have taken part in a similar study previously. Participants received a base compensation of 500 New Taiwan Dollar (NTD) for their participation in the study. A lot of artifacts in the EEG data led to the removal of two subjects from further analysis. Visual inspection suggested that the subjects had greater than 50% bad epochs in their data. The age range of the remaining 15 volunteers was between 21 and 38 years (eight male, seven female; mean: 29.875 years; SD: 5.31 years).

### 2.2. Experimental Design

**Figure 1:**
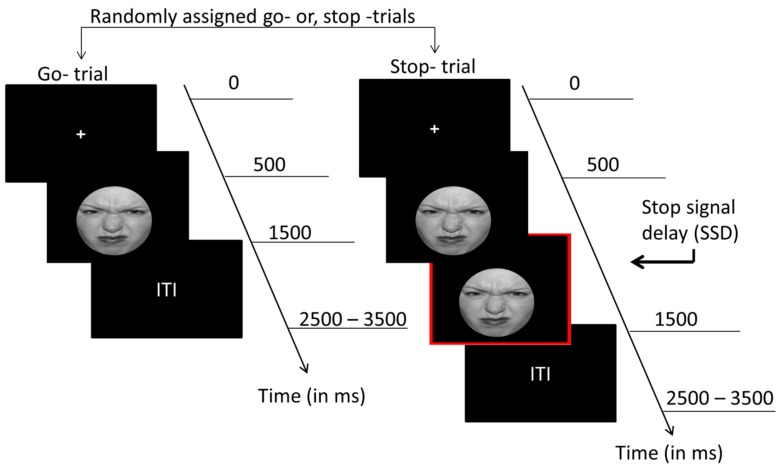
The emotional stop-signal paradigm (ESSP) employed in the current study. Subjects were presented disgusted, happy, and neutral faces to which they were asked to press a relevant button as quickly as possible. On seeing the stop signal, subjects were asked to refrain from pressing a button.

We performed the experiment in a sound-insulated dimly lit chamber where participants were asked to sit comfortably. We presented visual stimuli on a 24.4 × 18.3 cm computer monitor located 60 cm in front of them. The participants performed four sets of choice reaction tasks. The overall experiment design followed A-A-A-A block design. The emotional stop-signal paradigm (ESSP) (see Figure 1) employed in the current article had 168 trials per block divided into 120 go trials where the participants had to respond and 48 Stop trials where participants had to refrain from response if they observed a red border appear around the picture shown initially after 250 ms (stop-signal delay; SSD). Each trial started with a fixation cross for 700 ms followed by emotional picture presentation for 500 ms and variable inter-trial interval (ITI) between 1.5 and 2.5 s. For the trials with go stimuli, subjects had to press a button within 1 s of picture presentation identifying the type of face shown, that is, disgusted, happy, or neutral. Accordingly, subjects had to press “Z”, “M”, or “Space Bar” buttons on the keyboard for correctly identifying disgusted, happy, or neutral emotional faces. We adjusted the latency of the red border around the picture presented following the participant’s task performance to make the stop stimulus unpredictable. The participants were asked to respond as quickly as possible and not to worry too much about stop trials. We set the initial stop signal (red border around the picture) delay (SSD) to 250 ms. SSD was reduced by 50 ms on the subsequent stop trial if the participant was unable to stop successfully in the current stop trial and increased by 50 ms on the following stop trial if the participant was able to stop successfully in the ongoing stop trial (minimum SSD used was 50 ms; maximum SSD used was 450 ms). We calculated the stop-signal reaction times (SSRTs) by subtracting the mean of the SSDs from the median of the reaction times (RTs) on correct go trials for each condition. We divided the trials equally among three emotional conditions—happy faces, disgusted faces, and neutral faces. Thus, there were 160 go trials and 64 stop trials for each emotion condition. The faces were carefully hand-picked from the NimStim face database [64], which has been validated previously for emotion perception across various emotion types. We used a modified version of ESSP from a previously published fMRI study [29]. All the images selected were grey-scaled to a specific size (506 × 650 pixels; 96 dpi) and oval masked with a black background to avoid to impact of brightness, color, and other facial effects. We did this to prevent biased stimulus-driven response for subject’s choice response.

### 2.3. Recording and Analysis

#### 2.3.1. EEG Recording 

Electroencephalography (EEG) was recorded using 128 Ag/AgCl electrodes on the participant’s scalp (including six electrodes on the face) mounted on their head using a standard 10–20 system with a reference near the Cz electrode site. We controlled eye movements with the help of electrodes placed over and beside the eyes. Heart rate changes were accessed to be regressed out as covariates by placing one electrode over their index finger on both hands. We maintained electrode impedances below 20 kΩ and amplified signals using Neuroscan amplifiers with an analog bandpass of 0.1–100 Hz. We recorded EEG data at a sampling frequency of 1000 Hz. 

#### 2.3.2. EEG Analysis

EEG data were then re-referenced offline to the average of all electrodes, excluding electrooculograph (EOG), electromyograph (EMG), and electrocardiograph (EKG) electrodes. We applied a high-pass FIR filter at 0.1 Hz (cutoff frequency at −6 db was 0.05 Hz), and a low-pass FIR filter at 45 Hz (cutoff frequency at −6 db was 50.612 Hz). We performed all EEG preprocessing by using EEGLAB toolbox version 14.1.2 [65] and Matlab R2016a. Stimulus-locked epochs were extracted with a time window of 1400 ms before stimulus onset to 1500 ms after stimulus onset, using the pre-stimulus period from −1400 to −800 ms as baseline correction. Baseline correction was emotion and condition-specific, that is, disgust, happy, and neutral trials from the go condition had their baseline particular to the precise emotion type. We did not use a common baseline for all states because we assumed that happy, disgust, and neutral trials would elicit specific temporal differences in their epochs. Blinks were removed using independent component analysis. We removed epochs with missed responses and double responses from further analysis. Automatic epoch rejection removed trials with values outside −/+ 150 mV. After all rejection procedures, an average of 95 percent of epochs remained for further analysis. The response-locked epochs were extracted from 2400 ms before button press to 1000 ms after the button press. The response-locked trials baseline was set from −2400 to −1800 ms before the button press for disgust, happy, and neutral trials separately. Epoch rejection procedure was similar to stimulus-locked epochs.

#### 2.3.3. Group-Level Event-Related Spectral Perturbation (ERSP)

Event-related spectral perturbation (ERSP) analysis was used to look at theta frequency power at the medial frontal electrode site. We used the *std_precomp* function in EEGLAB to calculate group-level ERP and ERSP. We based our ERSP calculations on Fast Fourier transform (FFT) power spectrum of single-trial EEG data with the FFT power spectrum of a set of complex Morlet wavelets and taking the inverse FFT using *newtimef* function of EEGLAB. We computed ERSP-indices separately for every subject and experimental condition. We used a fixed window size of 256 samples (256 ms) across 116 frequencies from 2.0 to 30.0 Hz in the linear scale. Time-frequency plots were baseline corrected from −1400 to −800 ms before stimulus onset for stimulus-locked trials. We computed ERSP results for low frequency oscillations (LFO; 2–6 Hz) and beta frequency oscillations (12–17 Hz) in an early time window (200–350 ms for LFO power and 200–450 ms for beta power) owing to previous literature deeming these time-frequency power values relevant for emotion and response inhibition. The 2–6 Hz LFO frequency cluster was chosen instead of the delta and theta frequency band since we failed to observe a clear lower boundary at 4 Hz [66,67] for theta frequency, as seen in previous studies reporting motor conflict power changes in STN [43,51]. The experimental effect failed to demarcate sharp boundaries for delta and theta frequency power, so we combined 2–6 Hz as the LFO frequency cluster. The time windows were defined based on a priori knowledge of early sensory event-related potential (ERP) components like P1 and N1 dominating perceptual processing before 200 ms [68]. The time windows chosen for the LFO and beta frequency band more likely reflects the processing of emotion processing dominated by N2 or P3 ERP components [36]. For response-locked trials, baseline correction was performed on −2400 to −1800 ms before the button press. We computed ERSP results for LFO (2–6 Hz) and beta frequency oscillations (12–21 Hz) in the time window before button press (−300 to 0 ms for theta power and −400 to 0 ms for beta power). These time windows were chosen to reflect the accumulation of cognitive processes before a motor response. The response time ranges for beta and LFO differ since beta decrease was observed to be more prominent than LFO increase in the time post-stimulus presentation and preceding motor response. We had to adjust the time ranges based on group ERSP results for better comparison between emotion and neutral conditions in our data.

#### 2.3.4. Single-Trial ERSP

Single-trial ERSP was calculated using *pop_newtimef* function in EEGLAB. Average baseline power was derived from −1400 to −800 ms before stimulus onset and subtracted from relevant trials to create single-trial ERSP for LFO and beta oscillation separately for stimulus-locked trials. We obtained the response-locked ERSP power by baseline correction of −2400 to −1800 ms before the button press. The single-trial LFO and beta power were normalized by dividing their baseline value power in each trial. 

#### 2.3.5. Hierarchical Drift Diffusion Model (HDDM) Analysis

We used hierarchical Bayesian estimation as implemented in HDDM package version 0.6.0 [69] to look at trial by trial changes in parameters of the drift-diffusion model (DDM) defined formally by Ratcliff and colleagues [19]. We used RTs from go trials as done in previous stop-signal articles [58,59,60,70] in the DDM analysis across happy, disgust, and neutral conditions. In any trial, there were two possible responses for successful or failed emotion recognition (here we are choosing accuracy coding where 1 means subject was successful in identifying the correct emotional facial expression, and 0 meant the subject pressed incorrect button). We flipped error RTs to be negative. We used the deviance information criterion [71] for model comparison. To gain a deeper understanding of how visual affective information (disgust, happy, or neutral) affects choice RT (correct or incorrect emotion recognition), we defined three separate models and varied three DDM parameters of interest across the three visual conditions: evidence accumulation (drift rate, *v*), level of response caution (response threshold, *a*), and time needed for non-decision processes (non-decision time, *t*_0_). First, we inspect a set of stimulus-varying models by varying two or more DDM parameters across different stimulus conditions (Disgust stimulus, DS; Happy stimulus, HS; and Neutral stimulus, NS). Four models were created by varying (1) response threshold and drift-rate parameters, (M1), (2) drift-rate and non-decision times, (M2), (3) evidence accumulation and non-decision times, (M3), and (4) all three DDM parameters together (M4). Next, we modeled behavior data as a regression model by expressing DS and HS relative to NS condition. The regression model computed with behavioral data serves as a baseline to aid in comparisons to the regression models calculated with neural data as described below. Finally, we used two regression models to capture dynamics in frontal LFO and beta power varying with trial-by-trial reaction time. We did this separately for both stimulus-locked and response-locked trials. We estimated the regression coefficients between Fz activity decision thresholds in the same model, which was further used to determine the decision-making parameters themselves. In a given trial, we define the response threshold, *a*—

a = b_0_ + b_1_Stim + b_2_LFO + b_3_ LFO*Stim+ b_4_Betadecrease + b_5_Betadecrease*Stim, where Stim refers to the type of stimulus (DS, HS, or NS), LFO indicates the post-stimulus/pre-response increase in LFO, Betadecrease is the post-stimulus/pre-response decrease in beta power, and b_1–5_ are the estimated regression coefficients. 

For each model, HDDM obtains a sequence of samples, a Markov chain Monte Carlo (MCMC) from the posterior of each parameter. In the current manuscript, we generated 10,000 samples from the posteriors. To ensure that these MCMC samples come from a stationary distribution, we discarded the first 2000 samples as “burn-in”. For further modeling details, please see [59,60]. To prevent outliers, we discarded 5% of the data with the assumption that 5% of the data might not be generated by DDM process but instead by attentional lapses. We plotted a histogram of all the trials considered for HDDM analysis and found that RTs greater than 0.9 seconds and lower than 0.4 seconds constituted about 5% of the trials. To justify our approach, the model fitting before and after removal of 5% data is shown in Supplementary Figures S1, S2, and S3, respectively. We considered posterior probabilities ≥95% of the respective parameters being different than zero significant [44]. To compare different models, we assessed the difference between their respective deviance information criterion (DIC) values. Traditionally, DIC values >10 are considered as significant [44].

The HDDM code used to generate all these models is provided in the Supplementary Information to aid in better understanding of the models used in the study.

## 3. Results

### 3.1. Behavioral Analysis

During the successful go condition, reaction times were increased for neutral trials relative to happy (F(1, 14) = 20.022, *p* = 0.001, partial η^2^ = 0.589) but not disgust trials (*p* > 0.05) which is partially in line with dual competition framework [25]; happy stimuli captured more attentional resources relative to neutral stimuli. SSRT were increased for neutral trials relative to happy (F(1, 14) = 10.404, *p* = 0.006, partial η^2^ = 0.426) but not disgust trials (*p* > 0.05) which showed that subjects were better at inhibiting responses to happy stimuli relative to neutral stimuli [30]. We did not observe any significant findings in percentage choice error rates and failed RTs concerning go trials across emotions. Besides, the percentage of error rates in stop trials across happy and disgust emotion trials was not significantly different from neutral emotion’ trials. Finally, reaction times of failed stop trials, that is, stop respond times (SRTs) were faster than their corresponding go RTs but failed to reach significance for the disgust (*p* > 0.05) and happy (*p* > 0.05) emotions. However, differences in SRT and go RTs were seen for the neutral: (t(14) = 3.366, *p* = 0.005) emotion, which is in line with predictions made by race model [72]. We did not observe any effect of emotion on SRT, (*p* > 0.05). In this report, we did not test for differences in happy versus disgust stimuli since we were interested in comparing stimuli loaded with affect with neutral stimuli. We used neutral stimulus condition as a baseline for comparing the effects of disgust and happy emotion perception. All results in this report, therefore, focus on comparisons of happy versus neutral stimuli and disgust versus neutral stimuli.

### 3.2. Group Level ERSP Results across Conditions

#### 3.2.1. Stimulus-Locked ERSPs

**Figure 2:**
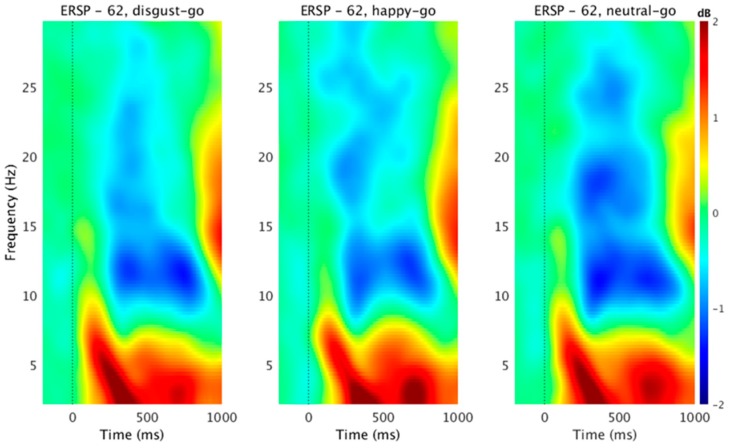
Event-related spectral perturbation (ERSP) associated with stimulus onset in the go condition. ERSP plot shows values averaged for the whole group (15 subjects) with insignificant masked reactions (green area) in decibels. Warm colors mean increase of power to the reference time interval; cold colors indicate a decrease. Left vertical lines represent the average time of the target stimulus onset. The dotted line is the target onset. *ERSP-62* corresponds to channel number. Here, we used the midline frontal ‘Fz” electrode for ERSP analysis.

We found a significant within-subject effect of emotion in go trials across LFO (F(2, 28) = 20.796, *p* < 0.001, partial η^2^ = 0.598), and beta frequency (F(2, 28) = 9.822, *p* = 0.001, partial η^2^ = 0.412) power. Looking deeper into the within-subject contrasts, the disgust emotion (F (1, 14) = 20.963, *p* < 0.001, partial η^2^ = 0.600) and happy emotion were significantly different from neutral emotion (F(1, 14) = 20.689, *p* < 0.001, partial η^2^ = 0.596) in the LFO power. For the beta power, the disgust (F(1, 14) = 5.282, *p* = 0.037, partial η^2^ = 0.274) as well happy emotions (F(1, 14) = 13.744, *p* = 0.002, partial η^2^ = 0.495) were significantly different from the neutral emotion. The group ERSP results for successful go conditions are shown in Figure 2. Emotional conditions were characterized by greater stimulus-locked LFO power (2–6 Hz) synchronization approximately 200 ms after stimulus presentation relative to the neutral condition at the midline frontal electrode site (“Fz”). We also observed greater beta power (12–17 Hz) desynchronization in neutral conditions relative to emotional conditions approximately 200 ms after stimulus presentation at Fz electrode site. These results overlap with previous evidence where frontal theta [73,74] and beta [74] power was shown to differentiate emotional stimuli activation from neutral stimuli.

#### 3.2.2. Response Locked ERSPs

**Figure 3:**
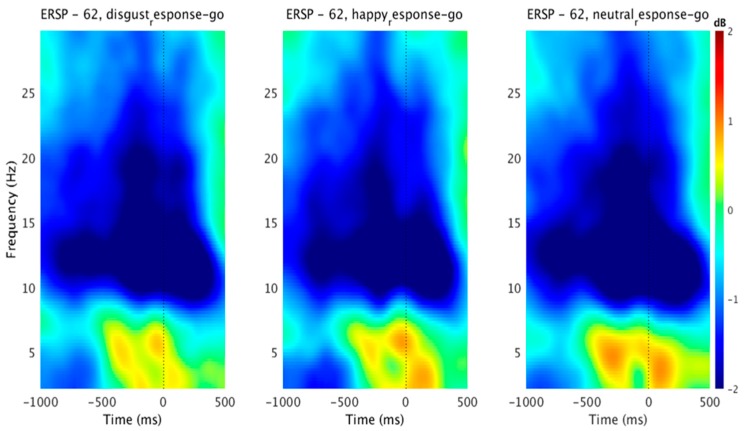
Event-related spectral perturbation (ERSP) associated with the response-locked trials in the go condition. The ERSP plot shows values averaged for the whole group (15 subjects) with insignificant masked reactions (green area) in decibels. Warm colors mean increase of power to the reference time interval; cold colors indicate a decrease. Left vertical lines represent the average time of the target stimulus onset. The dotted line is the target onset. *ERSP-62* corresponds to channel number. Here, we used midline-frontal ‘Fz” electrode for ERSP analysis.

We found a significant within-subject effect of emotion in go trials across LFO (F(2, 28) = 32.765, *p* < 0.001, partial η^2^ = 0.701), and beta frequency (F(2, 28) = 5.956, *p* = 0.019, partial η^2^ = 0.298) power. Looking deeper into the within-subject contrasts, the disgust emotion (F(1, 14) = 32.802, *p* < 0.001, partial η^2^ = 0.701) and happy emotion (F(1, 14) = 31.691, *p* < 0.001, partial η^2^ = 0.694) were significantly different from the neutral emotion in the LFO power. For the beta power, happy emotion (F(1, 14) = 6.191, *p* = 0.026, partial η^2^ = 0.307) was significantly different from the neutral emotion. Differences in beta power for disgust and neutral emotions failed to reach significance (*p* > 0.05). We have shown the group ERSP results for successful go condition in Figure 3. We characterized emotional conditions by LFO power (2–6 Hz) synchronization approximately 300 ms before response relative to the neutral condition at the Fz electrode site. We also observed greater beta power (12–21 Hz) desynchronization in neutral conditions relative to emotional conditions approximately 400 ms before response at the Fz electrode site. These results reinforce the role of response-locked theta and beta power in emotional stimuli recognition. The previous study found increased response-locked STN LFO power changes from 750 ms before response until response [45] for speed-accuracy trade-off during perceptual decision-making. Together with our stimulus-locked power results, these results could suggest that mPFC–STN transmit information in low frequency bands to support perceptual decision making. 

### 3.3. Exploring Trial-by-Trial Analysis of Correct Go Trials for Happy, Disgust and Neutral Conditions

**Figure 4:**
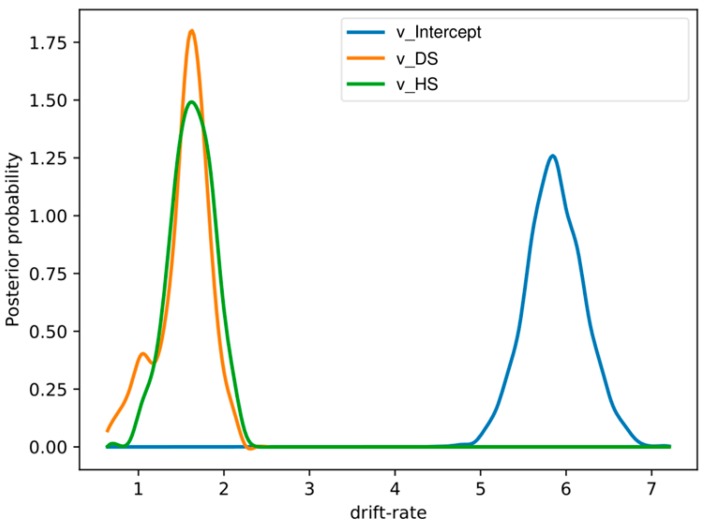
Group mean posteriors of within-subject drift-rate effects showing an increase in drift rates for the disgust and happy emotions relative to the neutral emotion. Here, we expressed DS and HS relative to the NS condition. Here, “v_.DS” and “v_ HS” represents drift rates in disgust and happy conditions relative to the neutral state.

The DIC values for the stimulus varying models are as follows (1) M1, (DIC: −10539.35), (2) M2, (DIC: −10547.72), (3) M3, (DIC: −10522.79), and (4) M4, (DIC: −10536.35). M4 had the lowest DIC value (best model) among the four models tested. However, M4 and M2 had comparable values when we correlated drift rates with SSRT and Go trials RT data. Since RT from go trials and SSRTs from stop trials were obtained by inspecting all the data as they are and not by comparing emotional conditions to neutral stimuli, we will use the best stimulus-varying model for comparison with behavioral data obtained from emotional response inhibition. These models assumed that different stimulus conditions (DS, HS, and NS) are completely independent of each other. However, this may not be true since a subject could perform well in emotional conditions relative to the neutral condition. The posterior probability of drift rates, response threshold and non-decision time for disgust (100% posterior probability) and happy (100% posterior probability) emotion were significantly different from neutral emotion in the within-subject regression model. We have shown the results in Figure 4. The DIC value for this within-subject regression model was −10318.81. 

### 3.4. Drift Diffusion Modeling with Behavioral Data

To ensure that the drift diffusion model accounted well for the data, we correlated drift rates from happy, disgust, and neutral conditions obtained from the stimulus-varying model M3 defined in Section 3.3 with reaction times from go-trials and SSRTs from stop trials. The drift rates for disgust, happy, and neutral stimuli are abbreviated as ‘v_subj_DS’, ‘v_subj_HS’, and ‘v_subj_NS’ respectively. The reaction times from go trials for disgust, happy, and neutral stimuli are abbreviated as ‘disgust_go’, ‘happy_go’, and ‘neutral_go’ respectively. The SSRT from stop trials for disgust, happy, and neutral stimuli are abbreviated as ‘disgust_stop_SSRT’, ‘happy_stop_SSRT’, and ‘neutral_stop_SSRT’ respectively. We have displayed the results in Table 1. Significant correlations of drift rate with SSRT and go-RT we see here are in line with previous stop-signal reports using DDM [60]. The results show that go and stop processes partly overlap with each other, and drift rate links these processes together when we consider response threshold and non-decision time parameters in our model. However, in contrast to a previous report [60], the response threshold and non-decision time parameters of our model were not significantly correlated with the drift rate for the emotional or neutral condition.

### 3.5. Exploring Trial-by-Trial Regression Analysis of ERSP Data with HDDM Parameters

#### 3.5.1. Stimulus-Locked Trials 

**Figure 5:**
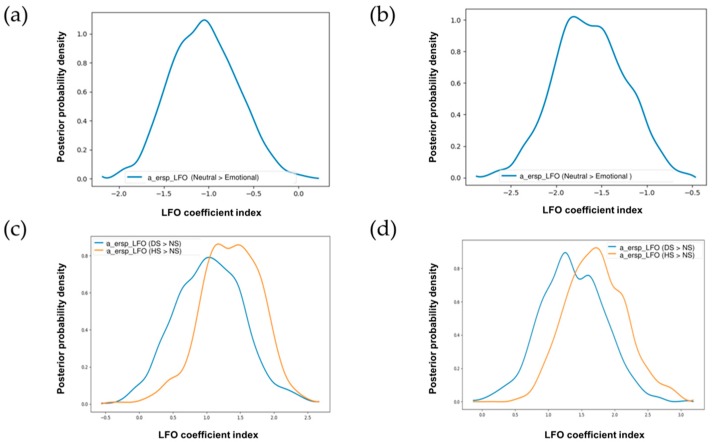
Single-trial ERSP associated with low frequency oscillatory (LFO) power for (**a**,**c**) stimulus-locked trials (**b**,**d**) response-locked trials. In the top two figures (**a**,**b**), posterior probability density reflects the decision threshold increase for emotional relative to neutral context. In the bottom two figures (**c**,**d**), posterior probability density reflects the decision threshold increase for the happy and disgust emotions relative to the neutral emotion. Here, “a_ersp_LFO (Neutral >Emotional)” represents the effect of trial to trial variations in medial frontal LFO for neutral condition relative to emotional condition while “a_ersp_LFO (DS >NS)” and “a_ersp_LFO (HS >NS)” represents the effect of trial to trial variations in medial frontal LFO for the disgust emotion relative to the neutral emotion and the happy emotion relative to neutral emotion respectively.

Next, we sought to compare if single-trial ERSP low frequency values in the early perceptual time window (200–350 ms) could predict changes in emotional versus neutral stimulus conditions across correct response trials in Go condition. Assuming that EEG LFO activity would be a predictor for a decision threshold in more difficult trials relative to lower difficult trials, we coded emotional stimuli (happy and disgust) as low difficult and neutral stimuli as high difficult. Effects of trial-to-trial adaptations in frontal LFO were found to decrease the estimated decision threshold: the regression coefficient was negative, and more than 99% of it was less than zero (Figure 5a). The result of low versus high difficulty for emotional relative to neutral context showed that LFO was indeed driven by emotion rather than a neutral setting which made the regression coefficient to be opposite to the hypothesized direction. The posterior probability showed that the strength of this was highly significant, which implied that frontal LFO was a significant predictor for emotional stimuli relative to neutral stimuli for correct go response. We also tested the effect of trial by trial adaptations of beta power in the early time window (200–450 ms) as a predictor for decision threshold. The assumption here was opposite to LFO activity. We assumed that high conflict neutral conditions would drive trial by trial beta power relative to low conflict emotional conditions. Effects of the trial-to-trial variations in the frontal beta were found to increase the estimated decision threshold: the regression coefficient was positive, and more than 92% of it was more than zero which implied that the frontal beta power was a better predictor for neutral stimuli relative to emotional stimuli for correct go response. However, the evidence was not significant. Previous reports on neural correlates of speed-accuracy tradeoff [44] have focused on an STN LFO power increase rather than beta power decrease. Our results point to a similar pattern. The sensorimotor conflict is associated with theta and beta oscillatory power in the STN [52]. Since our task involves emotions and response inhibition, we can classify our emotional response inhibition as a sensorimotor conflict task. However, we could not capture the trial-by-trial changes by beta oscillatory power but only by LFO power in the medial frontal cortex. 

We also modeled trial by trial changes in oscillatory power concerning variations in the stimulus. The model DIC value was lower (DIC: −10323.1928) than the model accounting for difficulty (DIC: −10294.1697), where we had grouped the happy and disgust emotions together. No other drift diffusion model parameters were varied in the two models to make model comparisons by based on the DIC values easier. Frontal LFO was a significant predictor for evidence accumulation for happy (100% posterior probability) as well as disgust (98% posterior probability) emotions in comparison to neutral trials (Figure 5c). The frontal beta decrease was not a significant predictor for the disgust (53% posterior probability) emotion or the happy emotion (59% posterior probability) in comparison to neutral trials. Contrary to previous report associating beta and theta oscillations to sensorimotor conflict, we did not find any evidence for a frontal beta decrease in ESSP. However, frontal LFO was related to emotional inhibition consistent with our hypothesis. Modeling emotional categories separately better explained the data as evident by lower DIC value. 

#### 3.5.2. Response-Locked Trials 

The model DIC value for stimulus-varying model (DIC: −10322.6942) was again lower than the difficulty modifying the model (DIC: −10286.0273). Frontal LFO was a significant predictor for evidence accumulation for emotional context relative to a neutral setting (100% posterior probability). We can see the results of difficulty varying model here (Figure 5b). We can see the results of the stimulus-varying model here (Figure 5d). Frontal LFO was a significant predictor for evidence accumulation for the happy (100% posterior probability) and disgust (99% posterior probability) emotions in comparison to neutral trials. The frontal beta decrease was a significant predictor for the disgust (96% posterior probability) emotion but not for the happy emotion (94% posterior probability) in comparison to neutral trials. Similar to the stimulus-locked condition, the response-locked condition also showed better model fit for separate emotional categories rather than emotions combined. Frontal LFO was deemed to be a significant predictor for both the happy and disgust stimuli. Interestingly, we found evidence in support of sensorimotor conflict in beta power decrease as well. However, we saw this effect for only disgust stimuli and not happy stimuli. We would guess that disgust stimuli drive beta power decrease rather than happy stimuli does when compared against neutral stimuli. Dual competition framework [23,25] postulates that negative emotion captures attentional resources than positive emotion. We would argue that this would lead frontal beta decrease to be a significant predictor for disgust stimuli relative to neutral stimuli and not for happy stimuli relative to neutral stimuli. 

## 4. Discussion

The current EEG study investigated how bottom-up sensory processes associated with emotional faces interact with top-down goal-driven inhibitory control and decision-making process. We found that emotional faces effect suppression of response as well as the equilibrium to respond swiftly and correctly. The behavioral data indicated that happy emotion trials were associated with slower reaction times and more efficient inhibition times as compared to neutral trials. Based on an alternate approach focused on decision components calculated from go trials, we observed a strong relationship between drift rate values, a proxy for the strength or speed of go processing and SSRTs, the behavioral proxy for efficient stopping. The findings from the current study try to link decision making components derived from go stimuli to stop trials. Our preliminary results suggest that stopping processes are directly related to the speed of responding to go stimulus, that is, slower the speed of responding to go stimuli reflects better stopping. Suppression of correct responses associated with stop trials was faster in the happy stimuli condition [75]. On a more general level, these results suggest that when emotional visual evidence is processed more quickly while neutral processes require additional processing time across go response and the inclination to stop a correct response. Our behavioral results as measured by RT data and SSRT did not support dual competition framework which postulates that negative affect should capture additional attentional resources as compared to neutral condition and this effect is obtained by faster RT and shorter SSRT for the disgust emotion relative to the neutral emotion. We believe that this could due to subjects not perceiving disgusted emotional faces as more different to neutral faces since faces were presented in a randomized fashion very quickly. We discuss the discrepancies of this effect in our analysis of neural data as measured by LFO and beta power.

The second goal of this study was to determine whether mid-frontal LFO and beta power changes were being modulated by visual affect in a trial by trial manner. Previous studies have demonstrated ERSP changes in delta and theta frequency for an early perceptual window in emotional relative to neutral conditions. We replicated the finding in our current study across go trials at the group level. Our present results add on to the literature that mid-frontal LFO contributes to response inhibition under emotional context for stimulus-locked trials as well as response-locked trials. Previous studies have reported enhanced delta and theta power for emotional conditions relative to the neutral condition [76,77]. Disgust and happy stimuli attract more attentional resources than neutral stimuli consistent with a previous report of an emotional go/no-go task using emotional stimuli [78]. The authors had shown that P2 ERP component helped distinguish disgust and neutral stimuli. A recent report looked at individual differences in emotion processing using HDDM. They concluded that “emotion effects of the tasks differed with a processing advantage for happy followed by neutral words in the lexical decision task and a processing advantage for neutral followed by happy and fearful faces in the gender categorization task” [79]. These results are similar to what we found in our ESSP using HDDM.

The role of frontal LFO in emotion recognition [73] and response inhibition [80] has been well studied in isolation but not so well as in conjunction, which is in emotional response inhibition. We took an alternate approach here to study mid-frontal LFO in go-trials rather than in stop trials as done in the literature. Mid-frontal LFO was greater for emotional relative to neutral condition for stimulus-locked trials. However, we observed the reverse trend for response locked trials. The response threshold parameter in our regression analysis supported this result showing that emotional and neutral trials could be distinguished well in ESSP. When we modeled the happy and disgusted emotions separately, we found that medial frontal LFO was a significant predictor for stimulus-locked as well as response-locked trials. Thus, medial frontal LFO modulated the trial-by-trial activity of ESSP in a context-dependent manner. Beta desynchronization modulated trial by trial activity in response-locked trials for disgust but not in the happy emotion. This provides new information that mid-frontal LFO and beta oscillations can predict evidence accumulation before the response in disgust emotion. Recent studies looking at N1 amplitudes and reaction times from go-trials in the stop-signal task have deemed changes in sensory N1 component as proactive stopping rather than reactive stopping [58]. The response-slowing phenomenon associated with emotional compared to neutral trials in the current study could also be an indicator of proactive inhibition. However, it is unlikely to classify our task as reactive and proactive inhibitory control since there was an interaction of sensory and inhibition processes. So, we will not speculate about the form of the stopping process involved in our ESSP. We suppose that interaction between proactive and reactive stopping processes eventually lead to inhibitory control in ESSP. Future studies using invasive intracranial electrodes or deep brain stimulation (DBS) could explore the role of proactive and reactive control in ESSP better as it is beyond the scope of scalp EEG. 

HDDM revealed behavioral differences between the disgust and happy emotions relative to neutral emotion concerning increase in drift rates, response threshold, and non-decision times. We suggest that emotion context improves evidence accumulation, level of response caution, and time required for non-decision processes. More importantly, the addition of neural data to HDDM improved the model fitting for stimulus-locked as well as response-locked trials. We suggest that mid-frontal LFO power predicts decision thresholds in response inhibition under emotional context. The role of mPFC theta in cognitive control is not a new result in itself. To the best of our knowledge, this is the first study using HDDM in emotional inhibition task to study the role of mPFC in emotional inhibition. The role of beta power in right frontal cortex has been found mainly in DBS and intracranial EEG studies on response inhibition and not much in scalp based EEG study like the current study. While we did not see the frontal beta decrease to be a significant predictor for emotion recognition in this study, it is hard to eliminate its role in emotional inhibition. Future studies might look at frontal beta and theta power simultaneously in larger samples to explore their role in emotional inhibition. 

HDDM is not the only existing modeling framework to perform hierarchical parameter estimation based on Bayesian statistics. A recent paper highlights an attractive complementary approach for performing Bayesian hierarchical parameter estimation [81]. Heathcote et al. provide an excellent framework to account for attention failures and choice errors in stop-signal data. Their framework complements the approach taken by HDDM. The dynamic models of choice (DMC) provide various criticisms with respect to standard methods accepted by HDDM. Both the tools are open-source and free to download (HDDM works on python code and DMC on R code). However, DMC is not a one-step solution to cognitive modeling. The authors themselves highlight their approach as being quite demanding for the user. We believe that HDDM provides models which are relatively easy to use and interpret while the DMC is for users who want greater control of their model estimates with a more hands-on approach. 

The current study does have a few limitations which must be kept in mind for future studies looking to replicate these effects in EEG data. Subjects were asked to perceive emotional faces as disgust, happy, or neutral in a short time without perhaps leading them to proper emotional recognition of the visual stimuli as done in several studies. Besides, we had a small sample size of 15 subjects. Future studies should look to improve on the sample size and try to replicate our findings. Lastly, we only focused on disgusted and happy faces. Thus, our study is not generalizable to emotional face perception measured by other facial expressions like anger, surprise, etcetera.

## 5. Conclusions

The current manuscript provides new evidence for dynamic modulation of sensory processing of go stimuli in response inhibition in emotional contexts using single-trial ERSP analysis. We characterized emotional go trials by higher LFO power relative to neutral go trials. We believe our work is in line with other work done on go trials exploring response inhibition. In emotional contexts, higher LFO power and lower beta power led to successful go responses. We also verified the results with drift-diffusion parameters which correlated well with behavior as well as neural data. Proactive stopping under emotional context in response inhibition could be useful for understanding patient populations with defective proactive response inhibition like Attention-deficit/hyperactivity disorder (ADHD) since their treatment also focusses on fronto–subthalamic circuits similar to ones dictated by the current study. 

## Figures and Tables

**Table 1:** Correlations between go-trial response times, stop-signal reaction time (SSRTs), and drift-rates.

		disgust_go	happy_go	neutral_go	disgust_stop_SSRT	happy_stop_SSRT	neutral_stop_SSRT	v_subj_DS	v_subj_HS	v_subj_NS
disgust_go	Pearson Correlation	1	0.980 **	0.897 **	0.604 *	0.545 *	0.361	−0.872 **	−0.924 **	−0.690 **
	Significance		0.000	0.000	0.017	0.036	0.186	0.000	0.000	0.004
happy_go	Pearson Correlation	0.980 **	1	0.870 **	0.600 *	0.587 *	0.336	−0.902 **	−0.960 **	−0.670 **
	Significance. (2-tailed)	0.000		0.000	0.018	0.021	0.221	0.000	0.000	0.006
neutral_go	Pearson Correlation	0.897 **	0.870 **	1	0.499	0.324	0.623 *	−0.793 **	−0.774 **	−0.730 **
	Significance. (2-tailed)	0.000	0.000		0.058	0.239	0.013	0.000	0.001	0.002
disgust_stop_SSRT	Pearson Correlation	0.604 *	0.600 *	0.499	1	0.637 *	0.237	−0.555 *	−0.576 *	−0.435
	Significance. (2-tailed)	0.017	0.018	0.058		0.011	0.395	0.032	0.025	0.105
happy_stop_SSRT	Pearson Correlation	0.545 *	0.587 *	0.324	0.637 *	1	0.203	−0.583 *	−0.625 *	−0.306
	Significance. (2-tailed)	0.036	0.021	0.239	0.011		0.469	0.023	0.013	0.268
neutral_stop_SSRT	Pearson Correlation	0.361	0.336	0.623 *	0.237	0.203	1	−0.346	−0.213	−0.585 *
	Significance. (2-tailed)	0.186	0.221	0.013	0.395	0.469		0.207	0.447	0.022
v_subj_DS	Pearson Correlation	−0.872 **	−0.902 **	−0.793 **	−0.555 *	−0.583 *	−0.346	1	0.937 **	0.764 **
	Significance. (2-0tailed)	0.000	0.000	0.000	0.032	0.023	0.207		0.000	0.001
v_subj_HS	Pearson Correlation	−0.924 **	−0.960 **	−0.774 **	−0.576 *	−0.625 *	−0.213	0.937 **	1	0.667 **
	Significance. (2-0tailed)	0.000	0.000	0.001	0.025	0.013	0.447	0.000		0.007
v_subj_NS	Pearson Correlation	−0.690 **	−0.670 **	−0.730 **	−0.435	−0.306	−0.585 *	0.764 **	0.667 **	1
	Significance. (2-tailed)	0.004	0.006	0.002	0.105	0.268	0.022	0.001	0.007	

** Correlation is significant at the 0.01 level (2-0tailed). * Correlation is significant at the 0.05 level (2-tailed).

## Supplementary Materials

The following are available online at https://www.mdpi.com/2076-3425/9/10/271/s1, Figure S1: Model fitting accessed by plotting probability density distributions of condition-specific RT data for disgust stimulus (DS): (a) data assumed to having no outliers (b) data assumed to having 5% outliers, Figure S2: Model fitting accessed by plotting probability density distributions of condition-specific RT data for happy stimulus (HS): (a) data assumed to having no outliers (b) data assumed to having 5% outliers, Figure S3: Model fitting accessed by plotting probability density distributions of condition-specific RT data for neutral stimulus (NS): (a) data assumed to having no outliers (b) data assumed to having 5% outliers.
